# Evidence for LINC1-SUN Associations at the Plant Nuclear Periphery

**DOI:** 10.1371/journal.pone.0093406

**Published:** 2014-03-25

**Authors:** Katja Graumann

**Affiliations:** Department of Biological and Medical Sciences, Oxford Brookes University, Oxford, United Kingdom; Iowa State University, United States of America

## Abstract

Sad1/UNC84 (SUN) domain proteins are a highly conserved family of inner nuclear membrane localised proteins in eukaryotes. One of their main functions is as key components of nucleo-cytoskeletal bridging complexes, in which SUN proteins associate with nucleoskeletal elements. In metazoans these are the lamins, which form a supportive structural network termed the lamina. Plants lack sequence homologs of lamins but have a similar nucleoplasmic structural network to support the plant NE. Putative components of this plant lamina-like structure are Little Nuclei (LINC) proteins, which bear structural resemblance to lamins and fulfil similar functions. This work explores the associations between AtLINC1, AtSUN1 and AtSUN2. AtLINC1 is recruited to the NE by SUN proteins and is immobilised therein. This recruitment and the immobile properties are likely due to AtSUN1/2-AtLINC1 protein interactions occurring *in planta*. In addition, the SUN N-terminus appears to play an important role in mediating these interactions. The associations between AtLINC1 and plant SUN proteins are a first indicator of how the nucleoskeleton may be anchored to the nuclear membrane in plants. Building on the previous characterisation of Klarsicht/Anc1/Syne1 homology (KASH) like proteins in plants, this study advances the identification and characterisation of nucleo-cytoskeletal bridging complexes in plants.

## Introduction

In plants and metazoans, the nuclear envelope (NE) is structurally supported by a nucleoskeletal meshwork underlying the NE on the nucleoplasmic side. In metazoans, this structure is termed the lamina as it consists of lamin proteins [1]. While plants lack sequence homologs of lamins, their nucleoskeletal structure is similar to the animal lamina [2], [3]. Both consist of similar sized filaments and appear to interconnect the nuclear pores [2]. Several plant-specific proteins are hypothesised to be components of this structure and functional homologs of lamins. One group of these proteins are the nuclear matrix constituent proteins (NMCPs). First identified in carrot, they are conserved in plants and have similar structural, biochemical and functional characteristics as lamins [4], [5]. In Arabidopsis, the NMCP homologs are referred to as Little Nuclei (LINC) proteins. While AtLINC1 and 4 are localised at the nuclear periphery, AtLINC2 and 3 are nucleoplasmic [6]. Other plant NMCP proteins are also localised at the nuclear periphery [4], [7]. AtLINC proteins are required for maintaining nuclei size and shape and multiple knock outs are lethal [6], [8].

In metazoans, various inner nuclear membrane (INM) intrinsic proteins bind to lamins and thereby connect the lamina to the NE. These INM proteins include the lamin B receptor (LBR) and lamin associated proteins (LAPs) as well as Sad1/Unc84 (SUN) domain proteins [1]. In plants, homologs of most of the well characterised metazoan NE proteins such as LBR and LAPs are not present [3], [9]. To date, only a few membrane intrinsic plant NE proteins have been identified, but they include the SUN proteins [10]–[12]. The two Arabidopsis SUN proteins AtSUN1 and AtSUN2 have been shown to have similar characteristics to metazoan SUN proteins including a similar domain layout with the highly conserved C-terminal SUN domain, localisation at the NE, specifically the INM, and the ability to interact with each other *in planta* at the NE [11]. While the molecular mechanisms of how nucleoskeletal components are anchored to the NE in plants remain to be identified, the plant SUN proteins are good candidates to mediate such anchorage. So far, AtSUN1 and AtSUN2 remain the best characterised plant INM proteins. More importantly though, substantial evidence from metazoan systems, where SUN proteins associate with lamins at the NE, suggest a similar role for plant SUN proteins.

In metazoans, SUN – lamina interactions are part of nucleo-cytoskeletal bridging complexes that span the INM and outer nuclear membrane (ONM) of the NE and directly connect cytoplasmic and nucleoplasmic structures [13]. The INM - localised SUN proteins not only associate with lamins but other nucleoplasmic components and chromatin. In the periplasm, the SUN domain mediates interactions with the Klarsicht/Syne1/Anc1 homology (KASH) domain of ONM – localised KASH proteins. These in turn complete the bridging by tethering cytoskeletal components including actin, microtubule and intermediate filament associated proteins [13]–[15]. The importance of these bridging complexes in various cellular and nuclear events such as chromosome movement, homologous pairing and nuclear movement and positioning have also been well documented in metazoan and yeast systems [13], [15]. Recent progress in the plant field is revealing similar NE bridging protein complexes. Both key membrane proteins – the SUN and KASH proteins – are present in plants and interact via the SUN and a plant-specific KASH domain [11], [16]. While both are required for maintaining nuclear shape, their plant-specific tethering of RanGAP to the nuclear periphery implies involvement of these complexes in nucleo-cytoplasmic transport [16], [17]. Recently, it was shown that the plant NE bridges are indirectly also linked to actin anchorage at the NE. The myosin XI-i was found to associate with WIT1, which in turn oligomerises with WIPs. This actin association is responsible for nuclei movement [18]. While this is the first NE-cytoskeletal association characterised in plants, further nucleoskeletal connections remain to be identified. In this paper, putative interactions between Arabidopsis SUN and LINC1 proteins are examined to provide first insights into the nucleoskeletal associations of nucleo-cytoskeletal bridging complexes in plants.

## Materials and Methods

### Constructs

The coding sequences (CDS) of AtSUN1 and AtSUN2 were cloned into pK7CWG2 for SUN-CFP fusions and pK7WGC2 for CFP-SUN fusions as detailed in Graumann et al [11]. For the N-terminal deletions, bp1-318 and bp1-312 were deleted from AtSUN1 and AtSUN2, respectively. For the SUN2 N-terminus construct SUN2^1–106^, the first 318 bp (106 aa) were amplified. AtSUN1ΔN, AtSUN2ΔN and AtSUN2^1–106^ CDS were cloned first into pDonr207 and then into pK7CWG2 using gateway technology. The plasmid pEG101::LINC1, containing the genomic sequence of LINC1 in frame with the CDS for YFP, was a kind gift from Eric Richards [6]. *Agrobacterium tumefaciens* strain GV3101 were transformed with the binary plasmids as described by Sparkes et al [19] and stored at −80C as glycerol stocks.

### Infiltration

Four to six week old *Nicotiana benthamiana* plants were used for transient expression with *A. tumefaciens* as detailed by Sparkes et al. [19]. *A. tumefaciens* carrying SUN-expressing vectors were infiltrated at an OD of 0.03 while *A. tumefaciens* carrying pEG101:LINC1 were infiltrated at an OD of 0.1.

### Confocal Imaging

At 3 days post infiltration (dpi), approximately 0.5 cm^2^ leaf sections were excised and mounted in water. A Zeiss LSM 510 confocal microscope with a x40 oil immersion lens was used to image the subcellular localisation of all fluorescent fusion proteins expressed. YFP fluorescence was excited by a 514 nm laser and emission captured in a 530–600 nm band pass filter. CFP fluorescence was excited by a 458 nm laser and emission captured by a 475–500 nm band pass filter. Z-stacks of a field of view were taken and fluorescent cells counted. For each sample, 50 field of view z-stacks were recorded and approximately 75 nuclei analysed. The sub-cellular localisation of AtLINC1-YFP was determined as either only nucleoplasmic, or nucleoplasmic with nuclear periphery accumulation, or nuclear periphery only. Expressing cells were categorised accordingly and presented as average percentage ± standard mean error; a Student T-test was used for statistical analysis.

### Acceptor Photobleaching Fluorescence Resonance Energy Transfer

At 3 dpi expressing leaf sections were used for apFRET to analyse protein interactions. A Zeiss LSM 510 META confocal system was used to image YFP and CFP simultaneously. Excitation lasers for both were kept below 5%. A constant region of interest (ROI) was selected, in which YFP and CFP fluorescence intensity was measured. Samples were scanned 5 times for an average pre-bleach value, then YFP bleached with a 100% 514 nm laser followed by 5 more scans. CFP fluorescence pre- and post-bleach was normalised and analysed as described previously [11]. The pre-bleach fluorescence was used as internal negative control. For each sample approximately 35 NE were analysed.

### Fluorescence Recovery after Photobleaching

At 3 dpi, expressing leaf sections were used for FRAP to analyse mobile behaviour of AtLINC1-YFP. Leaf sections were prepared as described and the same confocal system was used but with the x63 immersion oil lens. FRAP measurements and analysis were carried out as described previously [11], [16], [20], [21]. Briefly, a constant ROI of the NE was selected and fluorescence in this ROI was recorded before bleach and after bleach with a 514 nm argon laser set at 100% transmission. Fluorescence recovery was recorded for 30 sec post bleach. For each sample, approximately 35 NE were photobleached. Microsoft Excel was used for data normalisation and statistical analysis and GraphPad was used for curve fitting. Mobile fraction and half time were presented as average ± standard mean error.

## Results and Discussion

### AtLINC1-YFP Localisation is Affected by SUN Proteins

**Figure 1:**
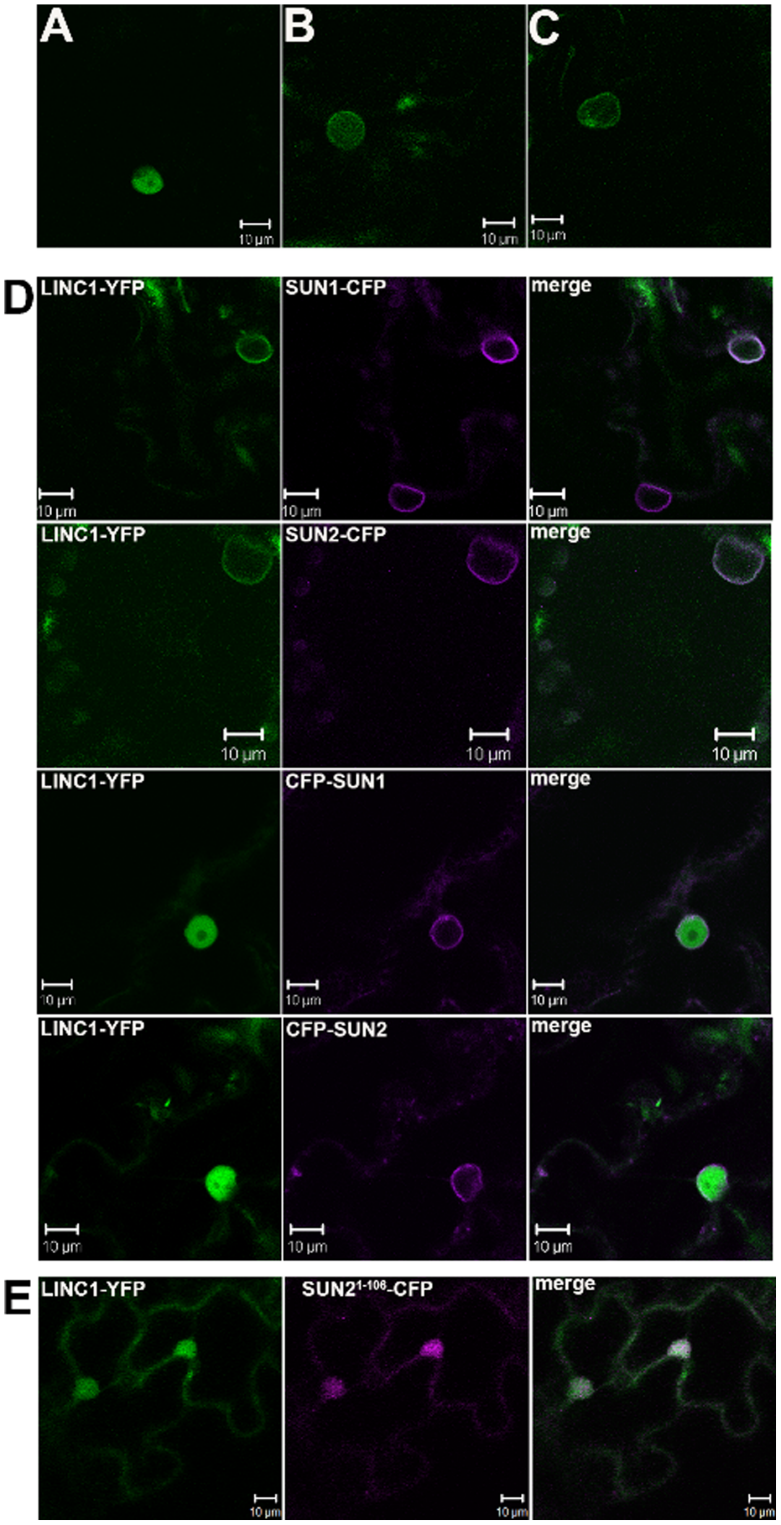
Subcellular localisation of AtLINC1-YFP; transient expression of AtLINC1-YFP results in three types of localisation: A) only nucleoplasmic, B) nucleoplasmic with nuclear periphery accumulation, C) only nuclear periphery; D) AtLINC1-YFP (green) remains localised at the nuclear periphery upon co-expression with AtSUN1-CFP and AtSUN2-CFP (magenta), which are present at the nuclear envelope. However, NE-localised CFP-AtSUN1 and CFP-AtSUN2 cause AtLINC1-YFP to remain nucleoplasmic indicating that the SUN protein N-terminus is involved in recruitment of AtLINC1-YFP to the nuclear periphery; E) AtSUN2^1–106^-CFP and AtLINC1-YFP co-localise in the nucleoplasm.

**Figure 2:**
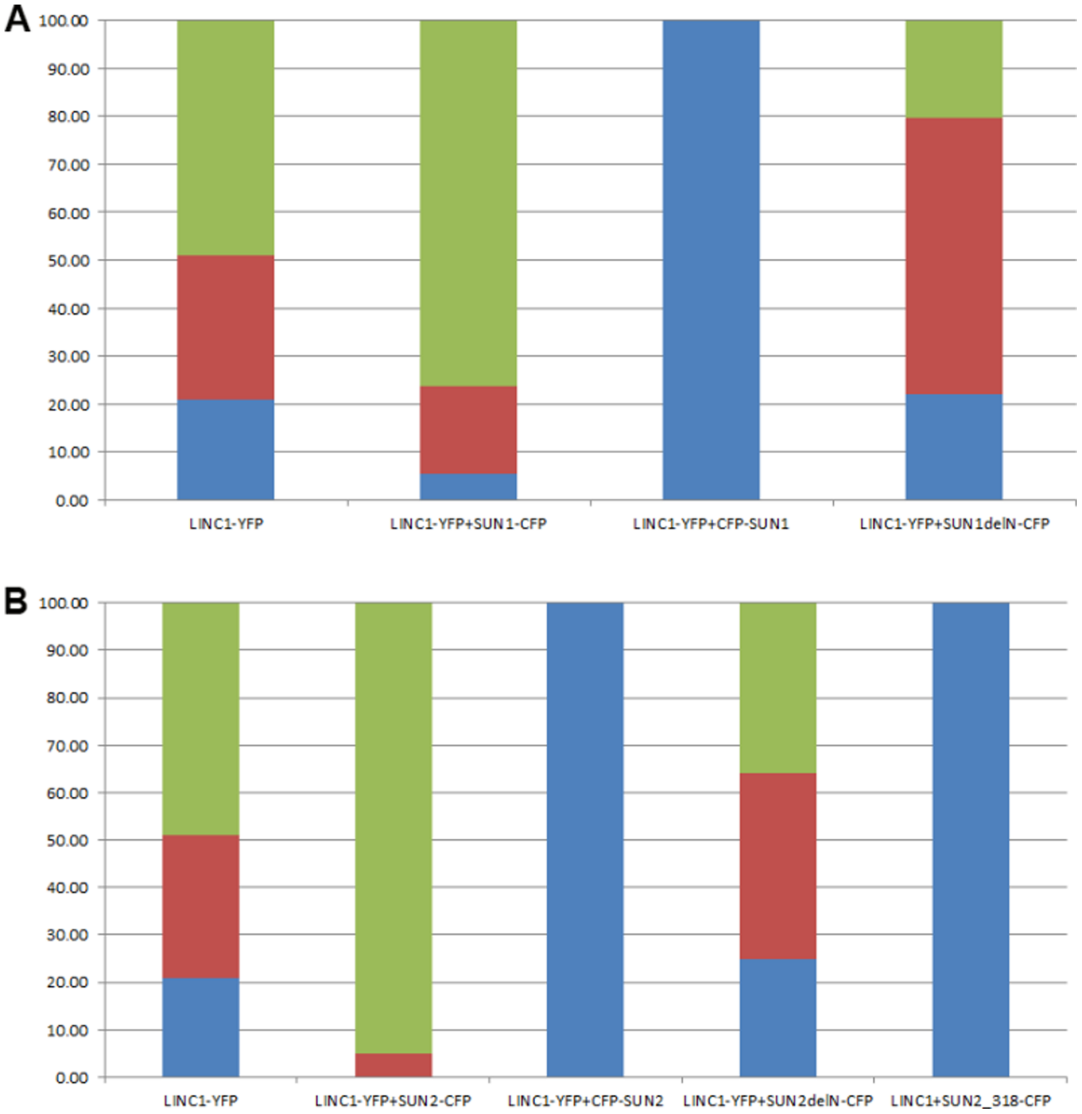
Changes in AtLINC1-YFP localisation; Percentage of expressing cells, in which AtLINC1-YFP is localised in the nucleoplasm only (blue), in both nucleoplasm and at the nuclear periphery (red), and only at the nuclear periphery (green). A) AtLINC1-YFP localisation upon co-expression with AtSUN1 constructs; B) AtLINC1-YFP localisation upon co-expression with AtSUN2 constructs; number of nuclei analysed = approx. 75 per sample.

Previously, yellow fluorescent protein (YFP) fusions of AtLINC1 (AtLINC1-YFP) were shown to localise at the nuclear periphery of stably transformed Arabidopsis plants [6], [8]. Here, transient expression was used to dissect subcellular localisation of AtLINC1-YFP in more detail. Three different subcellular localisations were scored for AtLINC1-YFP: 1. Nucleoplasmic, where the observed fluorescence was exclusively nucleoplasmic; 2. Nucleoplasmic and nuclear periphery, where the fluorescence was observed to be evenly distributed in the nucleoplasm but at the same time accumulated at the nuclear periphery; and 3. Nuclear periphery, where fluorescence was exclusively observed at the nuclear periphery. When AtLINC1-YFP was expressed by itself, all three forms of localisation were observed – in 20.83±8.17% of nuclei only nucleoplasmic, in 30.17±7.50% of nuclei both nucleoplasmic and accumulated at the nuclear periphery, and in the majority of nuclei (49.0±6.67%) exclusively on the nuclear periphery (Figure 1A–C, Figure 2, Table S1). This localisation pattern changed upon co-expression of full length AtSUN1 and AtSUN2 fluorescent fusions.

Co-expression with either AtSUN1-CFP or AtSUN2-CFP significantly increased the localisation of AtLINC1-YFP at the nuclear periphery (p<0.05). In 76.27±4.52% of AtSUN1-CFP co-expressing nuclei and 95.00±2.95 of AtSUN2-CFP co-expressing nuclei, AtLINC1-YFP was localised at the nuclear periphery alone (Figure 1D, Figure 2, Table S1). This suggests that SUN proteins may be involved in recruiting or anchoring AtLINC1 at the NE. Interestingly, co-expression with CFP-AtSUN1 and CFP-AtSUN2 had the opposite effect. In 100% of co-expressing cells, AtLINC1-YFP was present in the nucleoplasm with no accumulation at all at the nuclear periphery (Figure 1D, Figure 2, Table S1). The difference in AtLINC1-YFP localisation upon co-expression with either CFP-AtSUN1/2 or AtSUN1/2-CFP may be attributed to the functionality of the SUN protein fusions. Previously it was observed that N-terminal FP-SUN fusions have different levels of mobility compared to C-terminal SUN-FP fusions indicating that FP fusions interfere with binding interactions mediated by the termini of the proteins, which they are fused to [11]. As the N-terminus of SUN proteins is localised in the nucleoplasm, N-terminal CFP-SUN fusions may interfere with associations between SUN proteins and nucleoplasmic components, such as AtLINC1-YFP.

To investigate further the effect of the SUN N-terminus on AtLINC1 localisation, AtLINC1-YFP was co-expressed with C-terminal fused SUN proteins lacking the N-terminus (AtSUN1ΔN-CFP and AtSUN2ΔN-CFP) as well as with a construct containing the first 106 aa, the N-terminus, of AtSUN2 (AtSUN2^1–106^-CFP). While co-expression with CFP-SUNs completely abolished AtLINC1-YFP localisation at the NE, co-expression with the N-terminus deletions did not. However, AtSUN1ΔN-CFP and AtSUN2ΔN-CFP caused a significant reduction in AtLINC1-YFP presence at the nuclear periphery (p<0.05) (Figure 2, Table S1). In only 22.00±5.56% of AtSUN1ΔN-CFP co-expressing cells was AtLINC1-YFP at the nuclear periphery alone. This number was 25.00±6.10% for AtSUN2ΔN-CFP co-expressing cells. Both these values are significantly lower than for AtLINC1-YFP single expression indicating that indeed the SUN N-terminus is required for AtLINC1-YFP localisation at the NE. In both N-terminus deletion constructs, two nucleoplasmic amino acids remain before the start of the transmembrane domain. These residues may explain why some AtLINC1-YFP NE localisation was not completely abolished.

The role of the SUN N-terminus in AtLINC1-YFP localisation was also confirmed by co-expression of AtLINC1-YFP with the N-terminus of AtSUN2. AtSUN2^1–106^-CFP itself was nucleoplasmic (Figure 1E) and co-expression with AtLINC1-YFP caused 100% of cells to harbour only nucleoplasmic AtLINC1-YFP (Figure 1E, Figure 2B, Table S1). This mimics the CFP-SUN co-expression and indicates that the nucleoplasmic SUN2 N-terminus is sufficient to prevent AtLINC1-YFP recruitment to the NE.

### AtLINC1 Interacts with SUN Proteins at the Plant NE

To test putative protein interactions between AtLINC1 and the SUN proteins, acceptor photobleaching fluorescence resonance energy transfer was used. Protein interaction is detected when the CFP fluorescence increases (termed FRET efficiency or E_F_) significantly after the YFP bleach compared to an internal pre-bleach control. Such a significant CFP fluorescence increase was detected for AtSUN1-CFP (1.85±0.83%), AtSUN2-CFP (1.90±1.0%) and AtSUN2^1–106^-CFP (1.29±0.76%) indicating that the full length AtSUN1 and AtSUN2 as well as the AtSUN2 N-terminus can interact with AtLINC1-YFP at the NE and in the nucleoplasm (for AtSUN2^1–106^-CFP) (Table 1; Figure S1). No interactions with AtLINC1-YFP were detected for the N-terminus deletions of the SUNs as the E_F_ values remained similar to the pre-bleach control (−0.37±0.77% for AtSUN1ΔN-CFP and −1.34±1.09% for AtSUN2ΔN-CFP) (Table 1). The LINC1 interactions recorded here match with its localisation hence it is likely that AtSUN1-CFP/AtSUN2-CFP recruit AtLINC1-YFP to the NE by interacting with it. Likewise, nucleoplasmic AtSUN2^1–106^-CFP keeps AtLINC1-YFP in the nucleoplasm by binding. Abolishing LINC1-SUN interaction by deleting the N-terminus therefore may also be the reason for less AtLINC1-YFP recruitment to the NE.

**Table 1:** *In planta* interactions as detected by apFRET. FRET efficiency (E_F_) values with * are significantly different from control values (p<0.05) indicating protein interactions.

Sample	E_F_ (%)	Control E_F_ (%)
**SUN1-CFP+LINC1-YFP**	1.85±0.83*	−1.79±0.22
**SUN1ΔN-CFP+LINC1-YFP**	−0.37±0.77^#^	−1.53±0.21
**SUN2-CFP+LINC1-YFP**	1.90±1.0*	−1.47±0.28
**SUN2ΔN-CFP+LINC1-YFP**	−1.34±1.09^#^	−1.53±0.41
**SUN2^1^** ^–**106**^ **-CFP+LINC1-YFP**	1.29±0.76*	−1.29±0.20

E_F_ values with ^#^ are statistically similar to their control values (p>0.05) and significantly different to the * labelled E_F_ values (p<0.05) indicating no protein interactions; average ± standard mean error, n = 35.

### SUN Proteins Affect the Mobility of LINC1-YFP

Fluorescence recovery after photobleaching (FRAP) was used to observe the mobile behaviour of AtLINC1-YFP at the nuclear periphery and how it changes upon co-expression with SUN proteins. The mobile behaviour is an indicator of protein binding and is here used to underpin the protein interactions as identified by apFRET. A high mobile fraction indicates most of the protein is unbound and mobile whereas a low mobile fraction is due to strong binding interactions that immobilise it. Previously, this technique was used to characterise binding interactions between plant SUN and KASH proteins [16].

**Figure 3:**
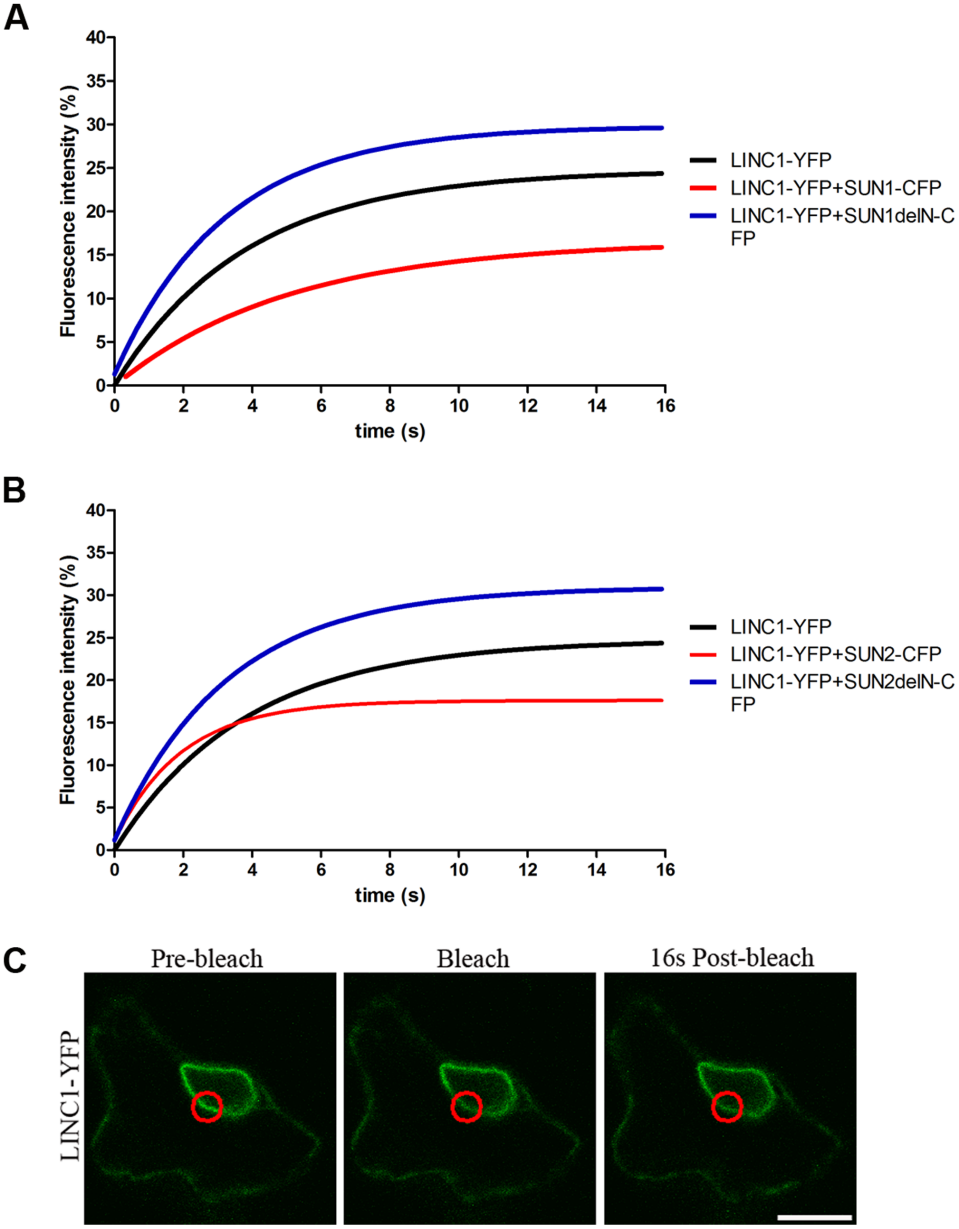
FRAP recovery master curves (relate to table 2); AtLINC1-YFP has a low recovery at the plant NE (black curves; A) AtLINC1-YFP mobility when co-expressed with AtSUN1 constructs; B) AtLINC1-YFP mobility when co-expressed with AtSUN2 constructs; full length AtSUN1-CFP/AtSUN2-CFP reduce the recovery of AtLINC1-YFP (red curves) but when the SUN N-terminus is deleted, AtLINC1-YFP recovery increases (blue curves); C) Representative image of FRAP experiment – LINC1-YFP fluorescence at the NE is bleached in ROI (red circle) and recovery monitored; size bar = 10μm.

When AtLINC1-YFP was expressed by itself, only 24.82±2.18% of the protein population at the nuclear periphery was mobile (Table 2, Figure 3). By comparison, NE protein mobile fractions have been recorded to vary between 40–90% [11], [16], [20], [21]. Thus, the low mobile fraction of AtLINC1-YFP indicates it is strongly anchored at the nuclear periphery, in the nucleoskeleton. Remarkably, the mobile fraction of AtLINC1-YFP decreased significantly further (p<0.05) upon co-expression with either AtSUN1-CFP or AtSUN2-CFP, with less than 20% of the AtLINC1-YFP population being mobile (Table 2, Figure 3). This demonstrates that the presence of the SUN proteins immobilises AtLINC1, due to tethering the protein to the nuclear periphery. Moreover, co-expression of either AtSUN1ΔN-CFP or AtSUN2ΔN-CFP caused the AtLINC1-YFP mobile fraction to recover to similar levels as when expressed by itself (Table 2, Figure 3). Hence, in the absence of the SUN N-terminus AtLINC1-YFP mobile fraction does not change but when the N-terminus is present (full length SUN) it declines. This implies the SUN N-terminus is associating with AtLINC1 and supports the binding interactions between SUN proteins and AtLINC1 as detected by apFRET. The half time of the AtLINC1-YFP mobile population differs in all samples, which may be due to parameters not linked to protein interactions. FRAP assays were not carried out for AtLINC1-YFP when co-expressed with AtSUN2^1–106^-CFP as the protein is not at the nuclear rim and therefore not comparable.

**Table 2:** Mobile behaviour of AtLINC1-YFP measured by FRAP; Mobile fractions labelled with ^#^ are statistically similar to each other (p>0.05) and indicate that LINC1-YFP is highly immobilised.

	Mobile fraction (%)	Half time (s)
**LINC1-YFP**	24.82±2.18^#^	3.49±0.49
**LINC1-YFP+SUN1-CFP**	19.58±1.12*	3.97±0.47
**LINC1-YFP+SUN1ΔN-CFP**	28.64±1.63^#^	2.07±0.20
**LINC1-YFP+SUN2-CFP**	18.43±1.64*	1.43±0.21
**LINC1-YFP+SUN2ΔN-CFP**	29.51±2.16^#^	2.13±0.21

Mobile fractions labelled with * are similar to each other (p>0.05) and significantly lower than ^#^ labelled values (p<0.05) indicating at further decrease in AtLINC1-YFP mobility; average ± standard mean error, n = 35.

### Exploring SUN-nucleoskeleton Associations in Plants

While plants lack sequence homologs of lamins, they contain a lamina-like nucleoskeletal structure, underlying the INM [2], [22]. Various filamentous nuclear proteins are hypothesised to be components of this structural network, such as filamentous plant proteins (FPPs) and NMCPs [3]. To date, mounting evidence from mass spectrometry of nucleoskeletal fractions, electron and light microscopy make a convincing case that NMCPs are components of this lamina-like structure in plants. These proteins are also structurally, biochemically and functionally similar to lamins [2], [5], [6], [8]. NMCP homologs have been identified in land plants with two groupings – NMCP1 and NMCP2 genes. In Arabidopsis, AtLINC1-3 are NMCP1 homologs while AtLINC4 is an NMCP2 homolog [5], [8]. The herein reported low mobile fraction of AtLINC1-YFP at the nuclear periphery strongly supports the case for AtLINC1 to be a component of the plant lamina-like structure.

This paper explores the relationship between AtLINC1 as a nucleoskeletal component and SUN proteins as NE components and hypothesised anchors of plant lamina-like elements. It presents first evidence of *in planta* protein interactions between SUNs and LINC1, the involvement of SUN proteins in recruiting LINC1 to the nuclear periphery and immobilising it there by binding. This study also highlights the importance of the SUN N-terminus in mediating these associations. Impairing the functionality of the N-terminus (CFP-AtSUN1/2) or deleting it, abolished or reduced AtLINC1 association with the NE. The N-terminus deletion also abolished interactions with SUNs. Conversely, the SUN2 N-terminus was sufficient to localise LINC1 to the nucleoplasm, likely due to binding interactions that occur between the two.

The relationship between SUNs and AtLINC1 as demonstrated here strongly suggests that in plants, members of the SUN domain protein family play an important role in nucleoskeletal anchorage at the nuclear periphery. As this is part of nucleo-cytoskeletal bridging across the NE, this study is a first indicator for the role of AtLINC1 in such bridging. Hence, a likely model for nucleo-cytoskeletal bridging in plants would consist of LINC1 as the nucleoskeletal component associating with the N-termini of SUN proteins, which in turn bind the VVPT domains of WIPs, the plant KASH proteins, via their SUN domains. On the cytoplasmic side, WIP-WIT complexes link to myosinXI-I as cytoskeletal element.

The importance of SUN proteins as nucleoskeletal anchors in plants also becomes apparent in the absence of protein homologs of other NE proteins such as LBR and LAPs, which play equivalent roles in metazoans. With only a handful of plant NE proteins identified to date, it appears likely that other, plant-specific NE proteins may additionally participate in nucleoskeletal anchorage. Further to AtLINC1, it is also expected that other nucleoskeletal components such as AtLINC4 and other NMCP homologs associate with NE intrinsic components. In fact, how the SUN-LINC1 associations are structured and regulated, remains to be explored. However, LINC-SUN associations at least appear to be involved in maintaining nuclear morphology and NE reformation. Knockout of SUN and LINC proteins causes nuclei to lose their cell-type specific shape and become rounded [6], [8], [16], [17]. This indicates that SUN-LINC1 associations, and hence NE-plant nucleoskeletal associations, are required for maintaining development-associated nuclear morphology. The anchorage may also play important roles during cell division, specifically NE reformation. FP fusions of the SUNs as well as the *Apium graveolens* LINC1 homolog, AgNMCP1, have a distinct localisation pattern during late anaphase and telophase. Both AtSUNs and AgNMCP1 first assemble on chromatin facing the spindle pole followed by further assembly around the sides of the decondensing chromatin and finally assembly on the chromatin that faces the division plate [7], [11], [17]. The SUN proteins were found to be immobilised in these membranes indicating functionality during the NE reformation event [21]. Interestingly, the WIPs also display this spatial-temporal distribution [23]. Thus, three separate components of putative nucleo-cytoskeletal bridging complexes – the ONM localised WIPs, the INM localised SUNs and nucleoskeletal LINC1– have the same distribution and might be involved, either independently or as part of a larger complex, in NE reformation in plant cell division.

### Conclusion

SUN-dependent NE localisation and immobilisation of LINC1 as well as LINC1-SUN interactions at the NE are strong indicators that SUN proteins are involved in nucleoskeletal anchorage at the plant NE. These results support an emerging plant NE bridging model, where SUN proteins in the INM associate with KASH-like WIPs in the periplasm via the SUN domain and with AtLINC1 in the nucleoplasm via the N-terminal domain. This also highlights similarities and differences of bridging complexes in plants, yeast and metazoans – their components can be both conserved across kingdoms (such as the SUNs) as well as specific structural and functional homologs with no sequence similarity and hence evolved separately.

## Supporting Information

Figure S1 **Representative image of apFRET experiment; AtLINC1-YFP fluorescence was bleached in ROI (red circle).** Upon bleaching, CFP fluorescence of AtSUN2-CFP increased; size bar = 10μm.
(TIF)

Table S1 **Localisation of AtLINC1-YFP values corresponding to** **Figure 2** **; average ± standard mean error, n = 75 nuclei.**
(DOCX)
