# Supplementary figures and images for: Evidence for LINC1-SUN Associations at the Plant Nuclear Periphery
Source: PLoS One. 2014 Mar 25;9(3):e93406. doi: 10.1371/journal.pone.0093406 (PMC3965549; doi:10.1371/journal.pone.0093406)

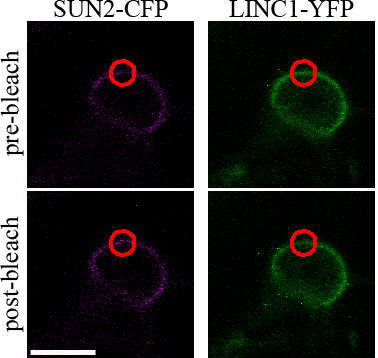

Supplement: Figure S1 — Representative image of apFRET experiment; AtLINC1-YFP fluorescence was bleached in ROI (red circle). Upon bleaching, CFP fluorescence of AtSUN2-CFP increased; size bar = 10μm. (TIF) [file pone.0093406.s001.tif]
