# Supplementary material for: Evidence for LINC1-SUN Associations at the Plant Nuclear Periphery
Source: PLoS One. 2014 Mar 25;9(3):e93406. doi: 10.1371/journal.pone.0093406 (PMC3965549; doi:10.1371/journal.pone.0093406)
Supplement: Table S1 — Localisation of AtLINC1-YFP values corresponding to Figure 2 ; average ± standard mean error, n = 75 nuclei. (DOCX) [file pone.0093406.s002.docx]

Table S1 Localisation of AtLINC1-YFP values corresponding to Figure 2

| **Samples** | **Nucleoplasmic %** | **Nucleoplasmic and periphery %** | **Periphery %** |
| --- | --- | --- | --- |
| **LINC1-YFP** | 20.81±8.17 | 30.17±7.50 | 49.00±6.67 |
| **LINC1-YFP+SUN1-CFP** | 5.33±1.79 | 18.40±4.12 | 76.27±4.52 |
| **LINC1-YFP+CFP-SUN1** | 100.00±0 | 0±0 | 0±0 |
| **LINC1-YFP+SUN1ΔN-CFP** | 22.00±5.56 | 57.67±6.62 | 20.33±5.58 |
| **LINC1-YFP+SUN2-CFP** | 0±0 | 5.00±2.95 | 95.00±2.95 |
| **LINC1-YFP+CFP-SUN2** | 100.00±0 | 0±0 | 0±0 |
| **LINC1-YFP+SUN2ΔN-CFP** | 25.00±6.10 | 39.00±6.74 | 36.00±6.71 |
| **LINC1-YFP+SUN2^1-318^-CFP** | 100.00±0 | 0±0 | 0±0 |
